# Rethinking AI-assisted writing instruction: feedback literacy scripts, calibration training, and student writing development

**DOI:** 10.3389/fpsyg.2026.1829268

**Published:** 2026-06-12

**Authors:** Zhe Dai

**Affiliations:** School of Humanities (School of Marxism), Zhejiang Institute of Communications, Hangzhou, China

**Keywords:** AI-assisted writing, feedback literacy, metacognitive intervention, self-assessment accuracy, writing quality

## Abstract

**Introduction:**

As generative AI becomes increasingly integrated into writing instruction, the central educational challenge is not only to provide feedback but also to help students interpret, evaluate, and use such feedback critically. This study examined whether two metacognitive interventions—a Feedback Literacy Script (FRAC) and an Assessment-Performance Calibration Activity (APCA)—could improve students' writing quality and self-assessment accuracy in AI-assisted writing.

**Methods:**

A 2 × 2 mixed factorial design was employed with 120 undergraduate English majors assigned to four conditions: regular AI use, FRAC only, APCA only, and FRAC+APCA. Across four writing task points, the study collected data on writing quality gain, self-assessment accuracy, overconfidence, effective feedback uptake, and revision depth.

**Results:**

The findings showed differentiated effects of the two interventions. FRAC had a stronger direct effect on writing quality, effective feedback uptake, and deep revision, suggesting that feedback literacy training primarily improved how students processed and enacted AI feedback. APCA showed the clearest effect on self-assessment accuracy and overconfidence reduction, indicating that calibration training more directly strengthened students' internal judgment. The combined intervention produced the highest gains in overall writing quality and the strongest retention after AI support was removed, but it did not outperform APCA alone in self-assessment accuracy.

**Discussion:**

These findings indicate that better writing and more accurate self-evaluation are related but distinct outcomes in AI-assisted writing. The study suggests that the educational value of AI-assisted writing depends less on feedback abundance itself than on whether learners can evaluate feedback, calibrate self-judgment, and transform external support into independent revision ability.

## 1 Introduction

Generative artificial intelligence is rapidly entering writing instruction and reshaping how students obtain, interpret, and use feedback (Mekheimer, 2025). Large language models such as ChatGPT and Claude can generate multi-level suggestions on vocabulary, grammar, structure, argumentation, and expression within seconds (Jovic et al., 2025). This increased availability of feedback expands support during writing, but it also changes what learners must do with that support. In AI-assisted writing, students need not only access to comments, but also the ability to judge their usefulness, evaluate their own understanding, and revise accordingly. For this reason, the effectiveness of AI-generated feedback depends heavily on feedback literacy and metacognitive awareness (Teng and Ma, 2024). The central issue is therefore no longer simple access to feedback, but whether students can regulate and use abundant feedback effectively (Zhan and Yan, 2025).

This shift has clear instructional implications. In traditional classrooms, teacher and peer feedback is often limited and delayed. In AI-supported contexts, by contrast, feedback is immediate, repeated, and easy to obtain. Although such accessibility may support revision, it may also create new risks if students lack the capacity to evaluate and monitor what they receive. They may accept suggestions without checking their validity, confuse receiving advice with solving a writing problem, or prioritize surface-level editing over substantive revision. AI feedback, then, does not automatically improve writing quality; its value depends on whether learners can filter, interpret, compare, and implement suggestions in a metacognitively informed way (Kasneci et al., 2023).

Existing research suggests that AI tools can improve linguistic accuracy, fluency, and writing efficiency in L2 and EFL writing contexts (Yan, 2023; Han and Li, 2024; Zhan and Yan, 2025). However, their effects on higher-order writing abilities remain mixed, particularly in relation to argument quality, transfer, and delayed retention. A major reason is that much of the literature has treated AI primarily as an input condition—AI vs. non-AI—rather than examining how students actually engage with AI-generated feedback during revision. As a result, less attention has been given to the internal processes through which students judge feedback credibility, reassess their own texts, and translate those judgments into revision. The key gap is therefore not only the limited use of process data, but also the lack of an explanatory framework linking feedback use, self-assessment calibration, revision behavior, and writing improvement.

This gap matters because AI feedback is not simply a faster version of teacher or peer feedback. Its credibility is not inherently stable; students must decide whether suggestions are accurate, relevant, and appropriate for the text at hand. Its outputs are also sensitive to prompts and interactional context, and may vary across occasions. Moreover, the speed and volume of AI feedback increase availability while also increasing the burden of selection, evaluation, and cognitive processing. As an interactive tool, AI may even encourage procedural dependence or anthropomorphic trust, which can weaken critical judgment of the feedback itself (Ranalli, 2021; Sullivan et al., 2023). Accordingly, a central pedagogical challenge in AI-assisted writing is how to support learners' monitoring and judgment in a feedback environment that is abundant, variable, and not always reliable.

Against this background, the present study argues that metacognitive support is as important as AI feedback itself in AI-assisted writing instruction (Teng, 2025). The study therefore focuses on two interventions that address different parts of the feedback-revision process. The first, the Feedback Literacy Script (FRAC), addresses the processing of external feedback. It helps students evaluate, select, and apply AI-generated suggestions more critically and effectively, in line with research showing that productive engagement with AI-generated feedback depends not merely on access to comments, but on students' feedback literacy and their ability to interpret and use feedback meaningfully (Yan and Carless, 2022). The second, the Assessment- Performance Calibration Activity (APCA), focuses on internal monitoring. It is designed to help students develop more accurate self-evaluation, reduce overestimation or underestimation, and improve the alignment between self-assessment and actual performance, drawing on research indicating that structured self-assessment can enhance learning while AI- or LLM-supported feedback may affect self-assessment accuracy in differentiated ways depending on learners' initial calibration (Heydarnejad, 2025). In brief, FRAC addresses how students work with external feedback, whereas APCA addresses how accurately they judge their own performance. Although these interventions are theoretically complementary, there is still limited evidence on whether they operate independently, reinforce one another, or yield diminishing returns in AI-supported writing contexts (Liebenow et al., 2025).

To address this issue, the present study uses a 2 × 2 mixed factorial design to examine the independent effects, interaction effects, and process mechanisms of FRAC and APCA in AI-assisted writing. The analysis goes beyond overall writing quality to include three related dimensions: within-task writing improvement, change in final draft performance across time, and process-oriented indicators such as feedback uptake, self-assessment accuracy, and revision depth. These dimensions make it possible to examine not only whether the interventions work, but also how they work. On this basis, the study addresses four research questions: whether FRAC and APCA improve writing quality and interact with each other, whether APCA improves self-assessment accuracy and reduces overconfidence after controlling for baseline differences, whether FRAC increases effective feedback uptake and promotes deeper revision, and whether the effects of these interventions transfer to new tasks and remain after AI support is removed.

## 2 Literature review

### 2.1 Applications of AI-assisted writing tools in education

Generative artificial intelligence is rapidly entering education, and writing instruction has become one of its most visible areas of application. Large language models such as ChatGPT and Claude can provide immediate feedback on student writing across multiple dimensions, including grammar, vocabulary, structure, argumentation, and writing style (Kasneci et al., 2023). Compared with teacher feedback and peer feedback, AI-generated feedback is more immediate, frequent, and personalized. It is therefore widely regarded as a promising tool for addressing the longstanding shortage of feedback in writing instruction. Existing studies generally suggest that AI-assisted feedback can improve student writing performance to some extent, particularly in linguistic accuracy, fluency, and the efficiency of local revision (Stevenson and Phakiti, 2014; Han and Li, 2024).

However, these positive findings are concentrated mainly in short-term interventions, single tasks, and surface-level language outcomes. As research has begun to examine argument quality, transfer, and retention after AI support is removed, findings have become less consistent. Some studies report improvements in final draft quality, whereas others show that gains in surface text quality do not necessarily lead to more accurate self-evaluation. In some cases, students who used ChatGPT became more likely to overestimate their writing ability or struggled to maintain their performance once AI support was no longer available (Grassini, 2023). These findings suggest that the educational value of AI-assisted writing is strongly process dependent and cannot be evaluated solely on the basis of final products.

More importantly, existing research often treats AI feedback as a single external input while paying limited attention to how students interpret, evaluate, and use that feedback. In practice, the main challenge is no longer whether students can access feedback, but whether they can use it effectively. Students may have difficulty judging the accuracy and relevance of AI suggestions. They may also find that highly immediate and dense feedback weakens their ability to assess their own actual writing level. As a result, they may prioritize surface-level, easy-to-handle changes while neglecting deeper revisions related to structure and argumentation (Nur Fitria, 2021). Survey-based evidence also indicates that students need more explicit guidance, particularly in how to use AI feedback critically and how to avoid overreliance on it (Baidoo-Anu and Ansah, 2023). Against this background, the present study focuses on two related but distinct process-level issues: how students process and adopt external feedback, and how they judge the quality of their own texts. These two issues correspond, respectively, to feedback literacy training and self-assessment calibration training.

### 2.2 Metacognition and self-regulated learning

Metacognition is commonly defined as individuals awareness, monitoring, and regulation of their own cognitive activity, a concept first systematically articulated by (Flavell 1979). Subsequent research on self-regulated learning has further shown that metacognition is a central mechanism through which learners actively manage task interpretation, strategy selection, process monitoring, and outcome revision (Flavell, 1979; Winne and Hadwin, 1998; Shen and Bai, 2024). This is especially important in writing, which is inherently a recursive process involving planning, translating ideas into text, monitoring, evaluating, and revising (Flower and Hayes, 1981; Tran and Ma, 2025).

Among the many indicators used in metacognitive research, self-assessment accuracy, or SAA, is one of the most common and operationally useful. It refers to the degree of alignment between learners judgments of their own performance and external evaluations, and can therefore be treated as a direct indicator of the quality of metacognitive monitoring (Panadero, 2017). Higher SAA generally means that learners are better able to identify genuine problems in their texts and are more likely to adopt revision strategies that address those problems effectively (Yan, 2020). By contrast, when learners consistently overestimate or underestimate their own performance, their subsequent revision decisions are more likely to diverge from task demands.

Research has repeatedly shown that self-assessment is often imperfect, especially when learners have limited ability, unclear evaluative standards, or face complex tasks (Andrade, 2019). This issue becomes more consequential in AI-assisted writing because students are exposed to many external suggestions at once. Learners who overestimate their texts may see less need for substantive revision, whereas those who underestimate their performance may accept suggestions indiscriminately. In this sense, SAA is not simply an additional outcome variable; it is a condition that shapes whether feedback can be used productively (Tang et al., 2024).

Recent scholarship has increasingly treated metacognitive accuracy as a process-related variable within self-regulated learning rather than merely an outcome, as learners' judgments about their own performance shape how they interpret tasks, allocate attention, and select revision strategies (Dinsmore et al., 2008; Dinsmore, 2017). Reviews by Panadero et al. (2018) and Yan (2023) similarly suggest that the effectiveness of formative assessment depends not only on the availability of external standards, but also on whether learners can internalize those standards into an actionable framework for self-judgment (Panadero et al., 2018; Yan, 2023). For this reason, research on SAA in AI-assisted writing should not focus solely on whether it improves. It should also examine how it interacts with feedback uptake, revision depth, and writing performance. Accordingly, research on SAA in AI-assisted writing should be connected to revision behavior and feedback use, rather than treated in isolation.

### 2.3 Feedback literacy and feedback uptake

Feedback literacy generally refers to learners ability to understand, evaluate, and use feedback effectively. This ability involves not only cognitive understanding of feedback content, but also emotional regulation in response to feedback and the capacity to convert feedback into subsequent action (Carless and Boud, 2018). Traditional feedback research has consistently shown that learning does not advance simply because feedback is provided. Progress depends on whether learners can translate feedback into revision decisions and concrete action (Boud and Molloy, 2013). In writing instruction, the crucial issue is therefore not whether feedback is given, but whether it is effectively taken up.

More recent work has further emphasized that effective feedback supports comparison, interpretation, and subsequent regulation, rather than merely indicating correctness (Panadero et al., 2024; Nicol and Rose, 2025). In actual writing practice, however, students often prioritize surface-level suggestions that are easier to implement and avoid more structural or argumentative feedback because these require greater evaluative effort. Research by Molloy et al. (2020) shows that the quality of feedback uptake is closely related to learners' metacognitive awareness, self-monitoring, and understanding of evaluative criteria (Molloy et al., 2020). This means that feedback literacy and SAA should be seen as related but distinct capacities: the former concerns the processing of external input, whereas the latter concerns the calibration of internal judgment.

The need for feedback literacy is especially pronounced in AI-assisted writing. Compared with teacher or peer feedback, AI feedback differs substantially in responsibility attribution, perceived authority, information density, and speed of generation. This means that students must do more than understand the suggestion itself. They must also judge whether it is trustworthy, whether it suits the current task, and whether it deserves revision effort. Winstone et al. (2022), in their discussion of discipline-specific feedback literacy, argue that feedback literacy is not an abstract, universal skill (Winstone et al., 2022). In writing contexts, this implies a need for explicit support that helps students decide which suggestions to adopt, which to postpone, and which to modify in light of task goals (Gozali et al., 2024; Henderson et al., 2025).

### 2.4 Calibration training and judgment accuracy

Calibration training is widely regarded as an important approach to improving self-assessment accuracy. Its core purpose is to help learners repeatedly compare their own judgments of performance with the actual quality of their work, thereby gradually developing a more stable internal understanding of evaluative standards (Panadero et al., 2017; Van der Kleij, 2024). Meta-analytic evidence shows that calibration training can significantly improve SAA and, under certain conditions, enhance self-regulated learning and academic performance. The value of calibration training lies not only in reducing judgment error, but also in helping learners form a more stable internal reference framework that supports better subsequent learning decisions.

Its effects in AI-supported settings, however, are unlikely to be straightforward. Brown et al. (2015) argue that the effectiveness of calibration training depends on the clarity of standards, the appropriateness of feedback timing, and the quality of reflective activity (Brown et al., 2015). In AI-assisted environments, these conditions may either be strengthened or undermined. On the one hand, AI can provide frequent external reference points that make it easier for learners to detect discrepancies in their own judgments. On the other hand, the immediacy and intensity of AI feedback may shift attention toward rapid text improvement rather than toward reflection on why judgment errors occurred. AI thus creates favorable conditions for calibration work, but it does not guarantee deeper evaluative engagement.

Andrade (2019) and Kostons et al. (2012) emphasize that calibration training works best when learners engage in relatively stable evaluative cycles with clear standards (Kostons et al., 2012; Andrade, 2019). Panadero and Alonso-Tapia (2013) further argue that accurate self-assessment develops gradually through sustained growth in standards understanding, self-monitoring, and strategic adjustment (Panadero and Alonso-Tapia, 2013). From this perspective, when learners are asked to perform both external feedback filtering and internal judgment calibration at the same stage, the two processes may either complement one another or partially interfere because of increased cognitive load and competition for attentional resources. In other words, FRAC and APCA should not be assumed to produce optimal outcomes across all dimensions simply because they are combined. A combined intervention may generate synergy in writing quality, but whether it also outperforms APCA alone in SAA remains an empirical question.

### 2.5 Research hypotheses

**Figure 1:**
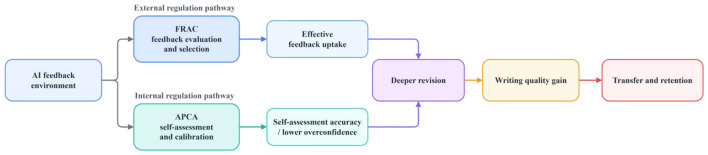
Conceptual model of AI-assisted writing support.

Based on the literature reviewed above, this study identifies two key abilities in AI-assisted writing: the ability to understand, evaluate, and use external feedback, and the ability to judge and calibrate the quality of one's own writing. FRAC primarily targets the first ability, whereas APCA mainly targets the second. Although the two interventions are theoretically complementary, their combined effects may vary across different outcomes. Figure 1 presents the conceptual model underlying these proposed relationships among the two interventions, process variables, and learning outcomes. The study therefore proposes the following hypotheses.

- H1: FRAC will significantly improve gains in writing quality. Students receiving FRAC are expected to achieve greater improvement in writing quality than students who do not receive FRAC, because feedback literacy training can support more effective use of AI feedback (Gozali et al., 2024).
- H2: Students receiving APCA are expected to show higher self-assessment accuracy and lower overconfidence than students who do not receive APCA, because calibration training helps strengthen internal evaluative standards (Panadero et al., 2018; Liebenow et al., 2025).
- H3: The combined FRAC + APCA intervention will produce greater gains in writing quality than either single intervention, but its advantage over APCA alone in self-assessment accuracy will be limited. Students receiving the combined intervention are expected to show stronger improvement in writing quality because they receive support for both external feedback use and internal judgment calibration. However, because APCA directly targets self-assessment accuracy, the combined intervention is not expected to produce substantially greater improvement in self-assessment accuracy than APCA alone (Yan and Carless, 2022; Henderson et al., 2025).
- H4: The effects of FRAC, APCA, and the combined intervention will extend to transfer and retention tasks. Students receiving FRAC, APCA, or the combined intervention are expected to maintain better performance than the control group in the transfer task and the retention task, suggesting more durable learning effects (Wang et al., 2025; Wirth et al., 2025).

## 3 Methodology

### 3.1 Research design

This study used a 2 × 2 mixed factorial design to examine the effects of two metacognitive interventions—the Feedback Literacy Script (FRAC) and the Assessment-Performance Calibration Activity (APCA)—on students' writing quality and self-assessment accuracy. The research design was grounded in a self-regulated writing perspective, with a focus on how students process external feedback and how they judge their own performance. Specifically, FRAC was used to guide students in understanding, filtering, and using feedback information, whereas APCA was used to help students calibrate the gap between their self-evaluations and actual performance. Based on the presence or absence of the two intervention factors, four groups were established: a regular AI group, a FRAC-only group, an APCA-only group, and a combined FRAC + APCA group. The regular AI group received only standard AI feedback and did not receive additional metacognitive training. The dependent variables included writing quality gain, self-assessment accuracy, effective uptake rate, and revision depth, while English proficiency, writing experience, prior AI use experience, and baseline writing ability were included as control variables.

**Figure 2:**
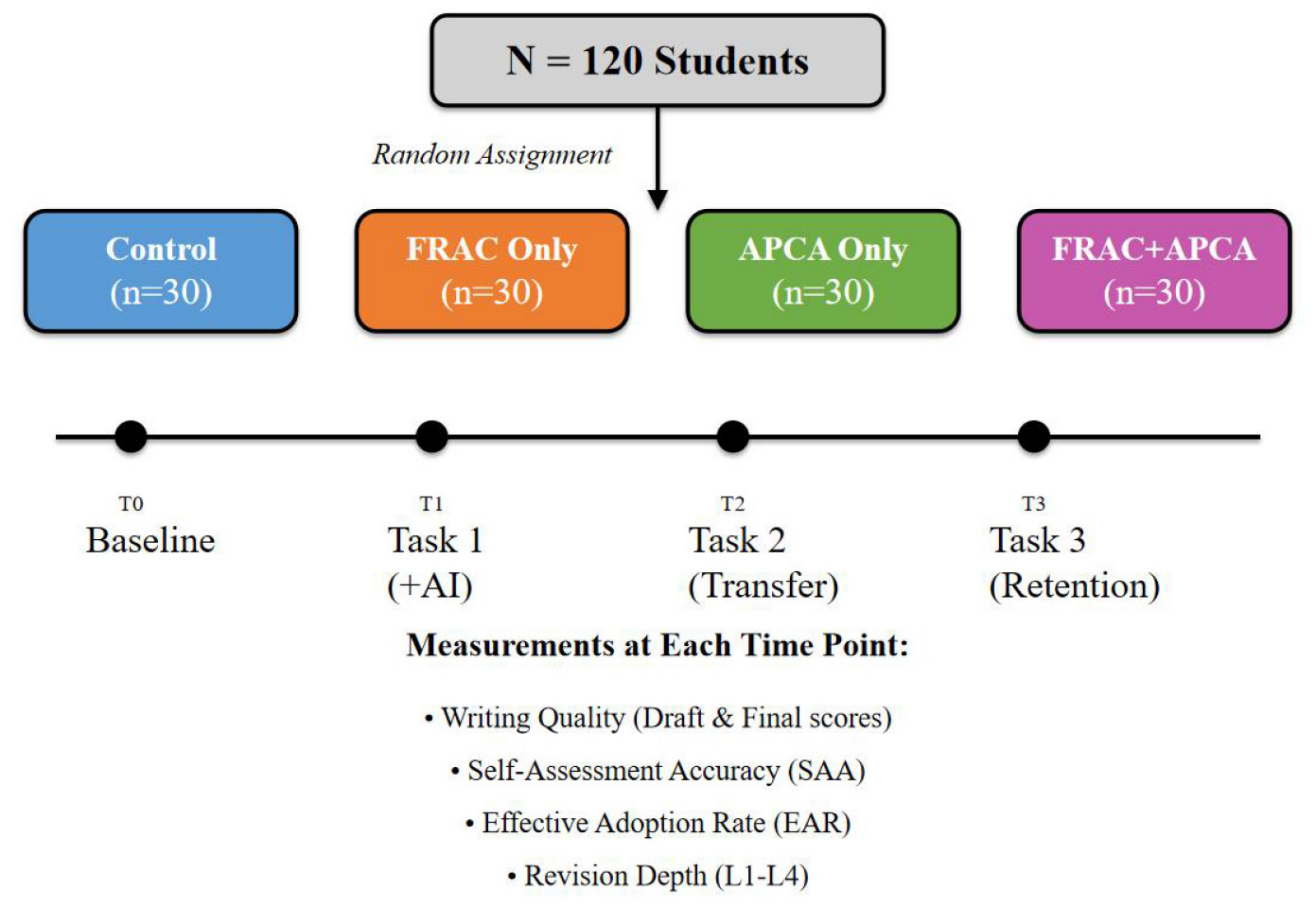
Experimental design and measurement procedure.

The study lasted six weeks and consisted of four time points. At T0, all students completed an initial draft, self-revision, and final draft under the condition of no AI support, which was used to establish the baseline. At T1, students completed an initial draft, received AI feedback, and revised their texts accordingly. T2 followed the same procedure as T1 but used a different writing topic in order to examine whether the intervention effects could be sustained in a new task. At T3, AI support was withdrawn again, and students were required to complete the writing task independently, allowing us to observe whether the earlier intervention effects could be retained without assistance. Based on this arrangement, T0 mainly served as a baseline reference point, the analysis of writing quality gain focused on T1 to T3, and comparisons across time points were based on final draft scores. The overall research procedure is presented in Figure 2.

In this study, Claude Sonnet 4 was used as the AI writing support tool because it can provide structured feedback across multiple dimensions. Feedback generation was conducted with a temperature setting of 0.4 to improve consistency. All students had access to AI feedback during T1 and T2, but the way they engaged with that feedback differed across groups. Students in the regular AI group could use AI feedback freely but received no metacognitive training. Students in the FRAC group were required to process AI feedback systematically through the Feedback Literacy Script. Students in the APCA group completed a calibration cycle consisting of self-assessment, feedback review, and comparison. Students in the combined group received both forms of training.

### 3.2 Participants

The study included 120 undergraduate English majors from the host institution, aged 19 to 22 years (M = 20.3, SD = 0.9). Female students comprised 78% of the sample, and male students comprised 22%. All participants had passed CET-6 or had attained an equivalent level of English proficiency and possessed basic academic writing skills. To be eligible, students had to be English majors, have completed at least one academic writing course, have basic experience with AI tools, have received no prior systematic training in AI-assisted writing, and be able to complete all tasks during the six-week study period. Participation was voluntary, and all students provided informed consent. Students with documented writing difficulties or learning disabilities, as well as those planning to participate in other writing training programs during the study period, were excluded.

**Figure 3:**
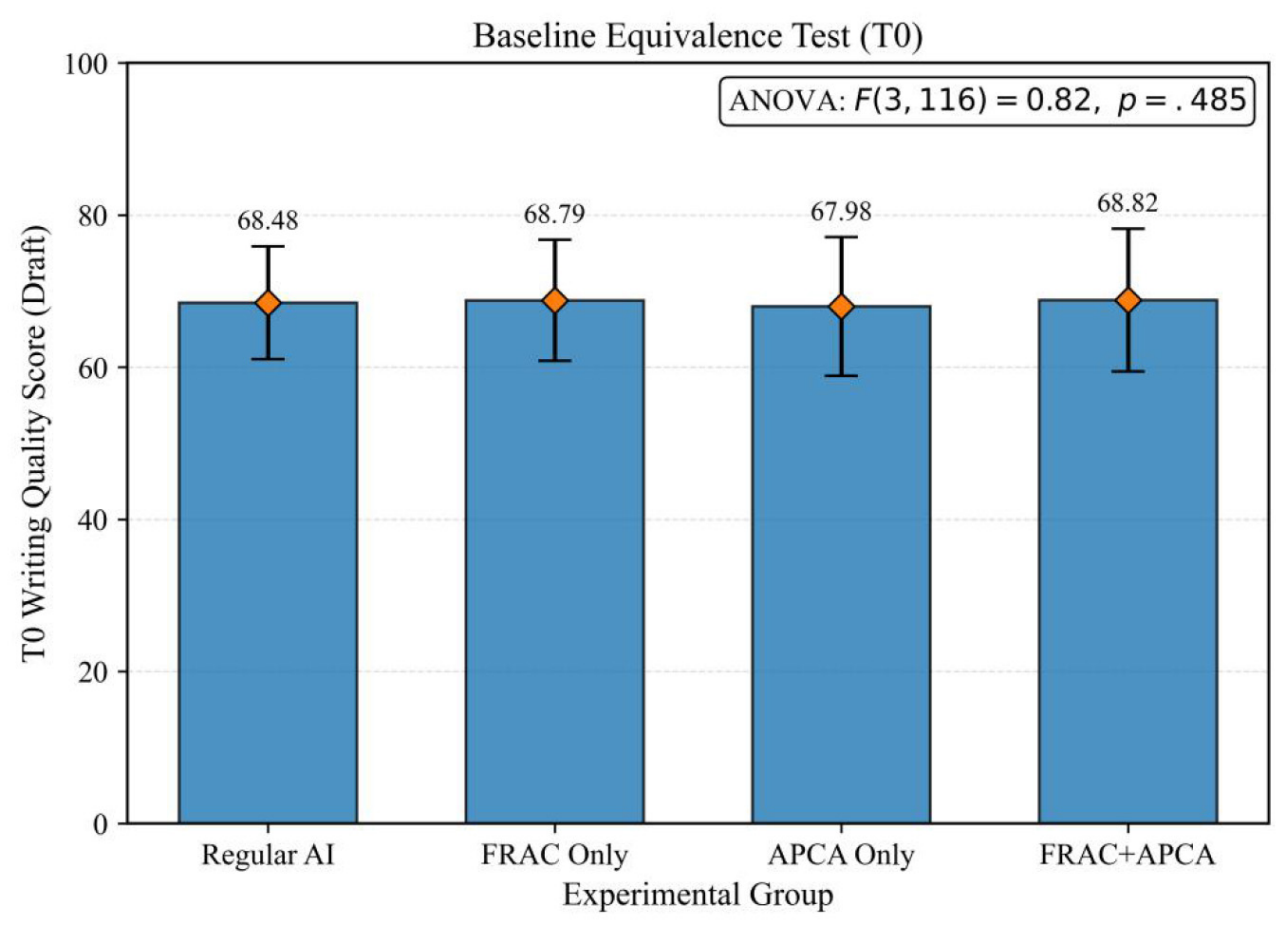
Distribution of T0 baseline initial draft scores across the four groups.

Participants were assigned to four groups of 30 through stratified randomization based on baseline writing ability, English proficiency, and prior AI use experience. Baseline equivalence tests showed no significant group differences in T0 initial draft scores, *F*(3, 116) = 0.82, *p* = 0.485, or in English proficiency and AI use experience, indicating good comparability across groups. The baseline means were 68.48 (SD = 7.43) for the regular AI group, 68.79 (SD = 7.95) for the FRAC group, 67.98 (SD = 9.11) for the APCA group, and 68.82 (SD = 9.40) for the combined group. However, because the raw T0 self-assessment accuracy values were not fully balanced, especially with lower error in the APCA group, later analyses of SAA were based mainly on baseline-adjusted models. The distribution of T0 baseline initial draft scores across the four groups is shown in Figure 3.

This study involved human participants and was approved by the university ethics committee of the host institution under Approval No. ZJIC-IRB-2025-EDU-371. Before the study began, all participants received a written information sheet explaining the research purpose, study procedures, the voluntary nature of participation, their right to withdraw at any time without penalty, and the academic use of the data. All participants provided written informed consent. Before data analysis, all personally identifying information was removed, and names were replaced with anonymous codes (S001–S120). Electronic files were stored on password-protected devices accessible only to the researcher.

### 3.3 Intervention procedures

The FRAC feedback literacy script was used to help students judge and use AI feedback more effectively. In the intervention design of this study, it mainly targeted students' processing of external feedback information. The emphasis was not on whether students accepted a particular feedback item, but on whether they could translate the feedback content into clear revision decisions. The script consisted of four steps: Filter, identifying the type of problem addressed by the feedback; Reason, judging whether the feedback was valid and relevant to the current task; Act, implementing the feedback to be adopted as a specific revision; and Check, reviewing whether the revision achieved the intended result. FRAC training was completed before T1 and lasted approximately 15 to 25 min, with brief reviews conducted before T1 and T2. Students in the FRAC group and the combined group completed decision sheets at T1 and T2 to record how they processed AI feedback. These records were included in the analysis as process data, and implementation was verified through submission rates, completeness checks, and classroom observations by the researcher. The return rate of materials exceeded 90% in each round.

The APCA self-assessment calibration training was mainly used to improve the accuracy of students' judgments of their own writing performance. The training was organized around a process of “self-assessment-comparison-adjustment,” requiring students to repeatedly compare their own judgments with external evaluations across multiple task rounds and revise their evaluative standards accordingly. The training included four stages: Anchor, reviewing sample texts at different quality levels to form basic evaluative reference points; Predict, estimating one's own score and confidence level after writing; Compare, identifying judgment bias by comparing self-ratings with teacher ratings; and Adjust, modifying subsequent evaluation strategies based on recurring tendencies toward overestimation or underestimation in previous rounds. APCA was implemented continuously from T0 to T3, with each round lasting approximately 10 to 15 min. In each round, students completed a structured calibration log recording their predicted score, confidence judgment, discrepancy from the teacher rating, and a brief reflection on recurring bias. The researcher reviewed these logs according to a unified feedback protocol and provided brief written comments for students to use in the next task. Both the APCA group and the combined group received this training. The combined group was included to examine whether external feedback-processing training and internal judgment-calibration training would produce additive effects when implemented together. Although AI support was no longer provided at T3, the self-assessment and comparison stages were retained to observe whether calibration performance could extend to an unsupported context. Implementation checks showed that the completion rate of calibration logs also exceeded 90% in every round.

### 3.4 Data collection

All writing tasks were academic argumentative essays to ensure comparability across tasks. At each of the four time points, students wrote 800 to 1000 words within 90 min. T0 focused on the impact of social media on university students' learning and was completed without AI support to establish baseline performance. T1 addressed online education vs. traditional education and followed an initial draft, AI feedback, and revision sequence. T2 examined the future of artificial intelligence in education and used the same procedure as T1, but with a different topic, to test transfer. T3 focused on the importance of lifelong learning in modern society and was completed without AI support to assess retention. All topics were piloted in advance to ensure comparable difficulty and relevance to students' background knowledge.

AI feedback was generated through a standardized procedure. Using a uniform prompt, Claude Sonnet 4 provided structured feedback on language accuracy, text structure, argument quality, and evidence support, with approximately 15 to 20 suggestions per essay. The research team reviewed the feedback for basic accuracy and appropriateness before it was returned to students. At T1 and T2, students received AI feedback within 24 h after submitting their drafts and submitted revised final drafts within the following 48 h. After each task, students also reported a predicted writing score on a 0 to 100 scale and a confidence level on a 1 to 5 scale, together with brief comments on their writing strengths, weaknesses, and perceptions of AI feedback.

Multiple sources of process data were collected to trace students' feedback use and revision behavior. Initial drafts and final drafts were collected at each time point. At T1 and T2, students in the FRAC group and the combined group also completed feedback decision sheets, in which they recorded how they handled each AI feedback item, why they made that decision, and what revision they carried out. Students in the APCA group and the combined group completed structured calibration logs across the four task rounds, recording their predicted scores, confidence judgments, discrepancies from teacher ratings, and brief reflections on recurring judgment tendencies. These materials were used to examine how students processed external feedback, calibrated their self-assessment, and translated feedback or self-evaluation into revision. The operational definitions and coding procedures for these measures are described in the following section.

### 3.5 Measures

SAA was one of the core measures in this study. It was calculated as the absolute difference between the score predicted by the student and the actual score assigned to the text, with smaller values indicating greater accuracy. To capture directional bias in self-evaluation, the study also computed a signed error score by subtracting the teacher-rated score from the student's predicted score; positive values indicated overestimation, whereas negative values indicated underestimation. Overconfidence was defined as a predicted score more than five points higher than the actual score, and an overconfidence rate was calculated accordingly.

Writing quality was rated independently by two experienced English teachers using a 100-point analytic rubric comprising four dimensions: content and argumentation, organization and structure, language and expression, and evidence and citation, with each dimension worth 25 points. In the content and argumentation dimension, raters examined whether the position was clear, whether supporting ideas were relevant and sufficient, and whether the development of arguments was logical. In the organization and structure dimension, raters assessed coherence across paragraphs, the effectiveness of the introduction and conclusion, and the sequencing of ideas. In the language and expression dimension, the main criteria included grammatical accuracy, lexical appropriateness, sentence variety, and overall comprehensibility. In the evidence and citation dimension, raters examined whether examples or supporting information were relevant, sufficiently integrated, and presented in a manner conventionally acceptable for the task.

Before formal scoring, the two raters completed a norming session using benchmark essays drawn from the same student population. They discussed scoring standards, compared sample ratings, and resolved discrepancies until a shared interpretation of the rubric was established. A second round of calibration was then conducted on pilot essays before formal scoring began. After standardized training and calibration, the two raters demonstrated high agreement, with an intraclass correlation coefficient (ICC) of 0.92 for the total score. Final writing scores were based on the average of the two ratings. Writing quality gain was defined as the difference between the final draft score and the initial draft score, and the main analyses focused on writing quality gains at T1, T2, and T3.

Feedback uptake was measured through adoption rate (AR) and effective adoption rate (EAR). AR referred to the proportion of AI feedback items adopted by students out of the total number of feedback items. EAR further assessed whether the adopted feedback addressed a genuine problem, was implemented appropriately, and actually led to improvement. A feedback item was coded as effective only when three conditions were met simultaneously: first, the original text contained an identifiable problem corresponding to the feedback; second, the student's implementation of the feedback was textually appropriate rather than mechanical or distortive; and third, the revised text showed a clear improvement relative to the initial draft. Any uptake that did not satisfy all three criteria was coded as adopted but ineffective. EAR coding was based on students' revision records and comparisons between initial and final drafts. In the FRAC group and the combined group, coders also consulted students' decision sheets to determine whether the feedback had been understood and enacted as intended. Two independent coders double-coded 20% of the sample, yielding Cohen's κ = 0.85.

Revision tracking was conducted by comparing initial and final drafts with difflib in Python. Revisions were then classified into four levels: L1, surface revision; L2, lexical- and sentence-level revision; L3, paragraph-level structural revision; and L4, content- and argument-level revision. L1 revisions included corrections to spelling, punctuation, formatting, and other minimal surface features that did not alter sentence meaning. L2 revisions involved adjustments to wording, sentence rewriting, or local grammatical restructuring, mainly aimed at improving expression at the sentence level. L3 revisions referred to changes at the level of paragraph organization, such as reordering ideas, adding topic sentences, improving transitions, or restructuring within-paragraph logic. L4 revisions involved substantive changes to claims, reasoning, the use of evidence, or the development of argumentation across the text. Because L3 and L4 more clearly reflected the reworking of discourse structure and content, they were categorized as deep revision, and their proportion in the total number of revisions was used as an indicator of substantive revision. Revision-depth coding was completed independently by two trained coders, and inter-rater agreement for revision-level classification reached Cohen's κ = 0.82.

### 3.6 Data analysis

Data analysis was conducted in four stages. First, all data were screened for completeness and consistency across the four task points. Because all 120 participants completed every stage of the study, no missing data treatment was required. Descriptive statistics, including means, standard deviations, proportions, and score distributions, were calculated for all major variables in order to summarize baseline characteristics and overall group patterns.

Second, baseline equivalence across the four groups was examined before the main analyses. Group differences in T0 initial draft scores were tested to determine whether the four conditions were comparable in baseline writing ability. Background variables, including English proficiency, prior AI use experience, and writing experience, were also checked to confirm the success of the stratified randomization. Because the raw T0 self-assessment accuracy values were not fully balanced across groups, especially with lower initial error in the APCA group, subsequent inferential analyses of self-assessment accuracy were based primarily on baseline-adjusted models rather than raw group means.

Third, the main outcome of writing quality gain was analyzed using a multilevel mixed-effects framework in which repeated observations at T1, T2, and T3 were nested within participants. The fixed effects included FRAC, APCA, time, and their interaction terms, so that the independent and joint effects of the two interventions could be estimated across tasks. Participant-level random effects were included to account for within-subject dependency across repeated measurements. Baseline writing ability and other relevant background variables were entered as control variables where appropriate. This model was used to test whether FRAC and APCA significantly improved writing quality gain and whether their effects remained stable across the transfer and retention tasks.

Fourth, self-assessment accuracy and related process variables were analyzed in light of their measurement characteristics. Self-assessment accuracy was operationalized as the absolute difference between students' predicted scores and teacher-rated scores, with smaller values indicating greater calibration accuracy. Given the baseline imbalance at T0, the analysis of self-assessment accuracy was conducted using baseline-adjusted models with T0 self-assessment accuracy as a covariate. Signed error was additionally examined to distinguish overestimation from underestimation, and overconfidence was treated as a categorical indicator based on whether the predicted score exceeded the actual score by more than five points. Group differences in effective adoption rate and revision depth were examined through between-group comparisons and descriptive profile analysis. Revision depth was coded into four levels (L1–L4), and the combined proportion of L3 and L4 revisions was used as the indicator of deep revision. When significant overall group differences were found, *post hoc* pairwise comparisons were conducted to clarify the source of those differences. In addition, descriptive effect sizes were reported for key between-group differences. For the overall four-group differences, η^2^ and ω^2^ were used as omnibus effect-size indices. For comparisons between focal groups, Cohen's d was reported to reflect the practical magnitude of the differences, thereby providing more interpretable information beyond statistical significance alone.

## 4 Results

### 4.1 Descriptive statistics

This study collected writing data from 120 participants across four time points, yielding a total of 480 writing samples. Data screening showed that all participants completed every task, and no missing data were identified. Table 1 summarizes the descriptive statistics for the core measures across the four experimental groups. Overall, the four groups showed similar T0 baseline initial draft scores, which supports the comparability of baseline writing ability. However, the raw T0 means for self-assessment accuracy were not fully balanced, with the APCA group starting from a lower error level. Therefore, subsequent significance testing for self-assessment accuracy is based primarily on baseline-adjusted models.

**Table 1:** Descriptive statistics for the core measures across the four experimental groups.

Group	T0 baseline initial draft score M (SD)	Mean writing gain at T1–T3 M (SD)	SAA absolute error M (SD)	Overconfidence rate	EAR M (SD)	Deep revision proportion (L3 + L4)
Regular AI group	68.48 (7.43)	3.10 (1.95)	4.17 (3.22)	23.3%	—	28%
FRAC group	68.79 (7.95)	6.10 (2.04)	4.40 (3.42)	32.5%	0.68 (0.12)	45%
APCA group	67.98 (9.11)	4.56 (1.98)	2.25 (1.87)	5.0%	—	32%
Combined group	68.82 (9.40)	7.92 (2.04)	4.57 (3.57)	30.0%	0.65 (0.14)	48%

At the descriptive level, the four groups showed different strengths across the two key outcomes. For writing quality gain, the regular AI group showed the smallest improvement (M = 3.10, SD = 1.95), followed by the APCA group (M = 4.56, SD = 1.98), the FRAC group (M = 6.10, SD = 2.04), and the combined group, which achieved the highest gain (M = 7.92, SD = 2.04). This pattern suggests that FRAC was more closely associated with writing improvement, and that the combined intervention showed the strongest descriptive advantage in writing quality.

By contrast, the best descriptive performance in self-assessment accuracy was found in the APCA-only group rather than the combined group. The APCA group had the lowest mean absolute error (M = 2.25, SD = 1.87) and the lowest overconfidence rate (5.0%). The regular AI group, FRAC group, and combined group showed higher SAA absolute error and higher overconfidence rates. Because baseline self-assessment accuracy was not fully balanced across groups, these descriptive results should be interpreted together with the baseline-adjusted model reported below. Overall, the descriptive results suggest a differentiated pattern: the combined intervention was most beneficial for writing quality, whereas APCA alone showed the strongest descriptive advantage in self-assessment accuracy and overconfidence reduction.

### 4.2 Between-group differences in writing quality gain

**Figure 4:**
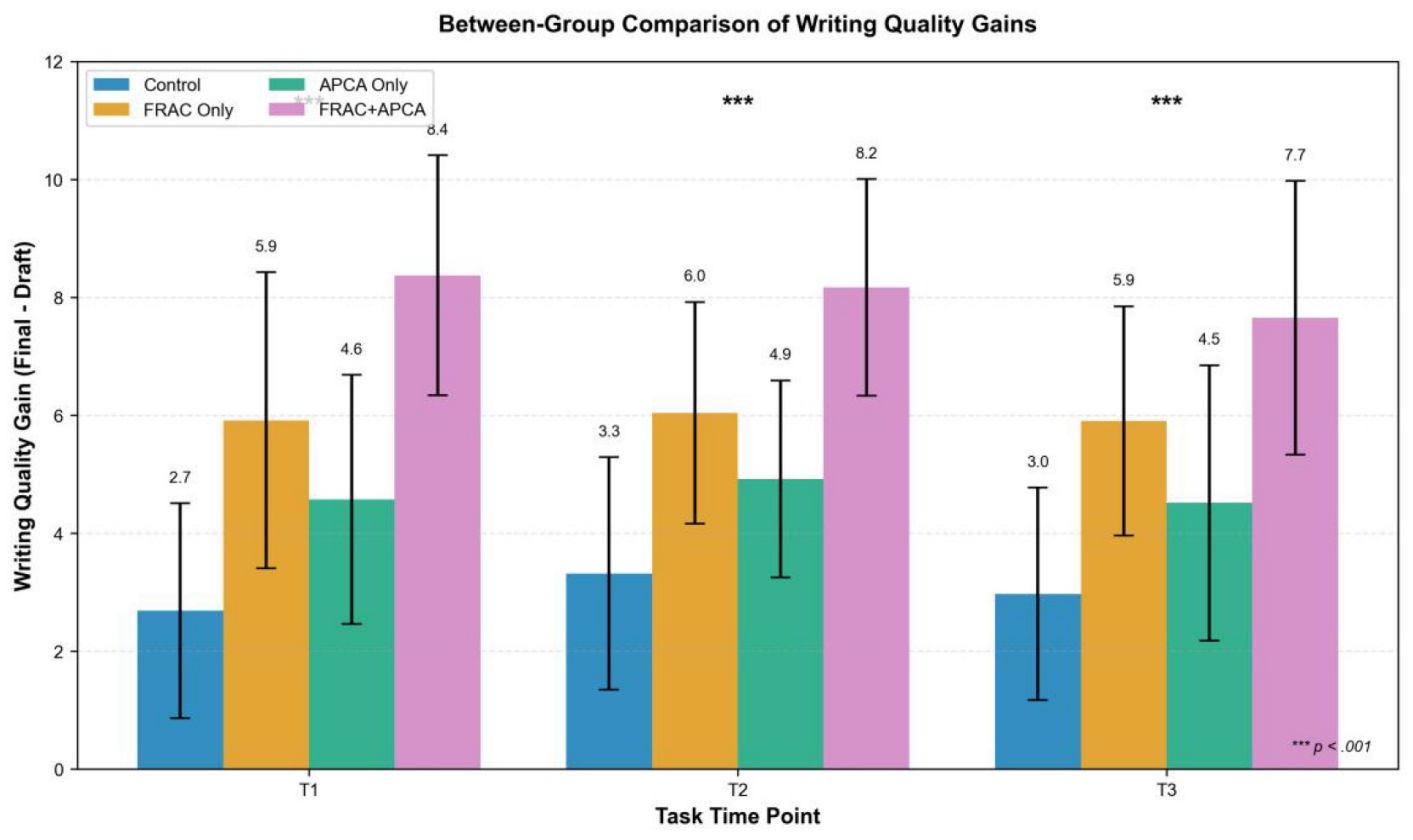
Comparison of writing quality gains across the four groups from T1 to T3. ^***^p < 0.001.

As shown in Figure 4, the four groups displayed a clear and stable pattern of writing quality gain across T1, T2, and T3. The combined group consistently achieved the highest gains, followed by the FRAC group, the APCA group, and the regular AI group. Across the three rounds, the combined group gained approximately 8 points, the FRAC group approximately 6 points, the APCA group approximately 4.5 to 5 points, and the regular AI group approximately 3 points. This pattern suggests that the combined intervention showed the strongest descriptive advantage in improving writing quality, while FRAC alone appeared to be more closely associated with writing development than APCA alone.

The group ranking remained largely unchanged across the three task points. At T3, when AI support was removed, the combined group and the FRAC group still maintained higher writing gains than the APCA group and the regular AI group. Although the T3 values were slightly lower than T2 in most groups, the overall pattern remained stable, suggesting that the intervention effects, especially those associated with FRAC and the combined intervention, were not limited to the AI-supported revision tasks.

### 4.3 Changes in self-assessment accuracy

**Figure 5:**
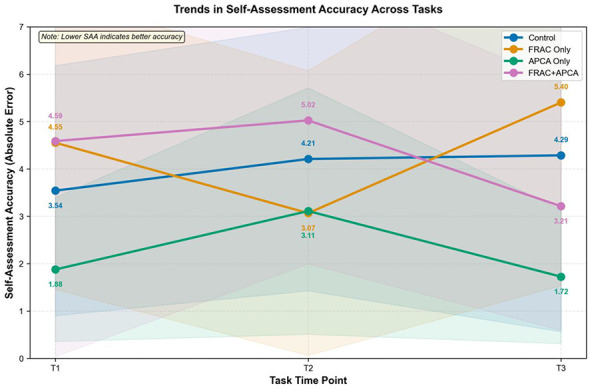
Trends in self-assessment accuracy across the four groups.

Self-assessment accuracy was measured by absolute error, with lower values indicating greater accuracy. Because the APCA group had lower raw error at T0, the main analysis used a baseline-adjusted model controlling for T0 SAA. Therefore, the descriptive trends shown in Figure 5 should be interpreted together with the baseline-adjusted analysis.

As shown in Figure 5, the APCA group generally showed the lowest self-assessment error across the three task points, although its error increased at T2 before decreasing again at T3. The regular AI group showed higher error than the APCA group across T1 to T3. The FRAC group fluctuated across tasks, with lower error at T2 but higher error at T3. The combined group also showed relatively high error at T1 and T2, but its error decreased at T3 after AI support was removed. Overall, the combined group did not show better self-assessment accuracy than the APCA-only group.

These descriptive patterns suggest that APCA was more closely associated with improved self-assessment accuracy than FRAC or the combined intervention. In particular, the APCA group showed the lowest error at both T1 and T3, whereas the combined group did not consistently outperform the APCA-only group. This pattern indicates that combining feedback literacy support with calibration training did not necessarily produce additional descriptive advantages for self-assessment accuracy.

### 4.4 Between-group comparison of effective adoption rate

**Figure 6:**
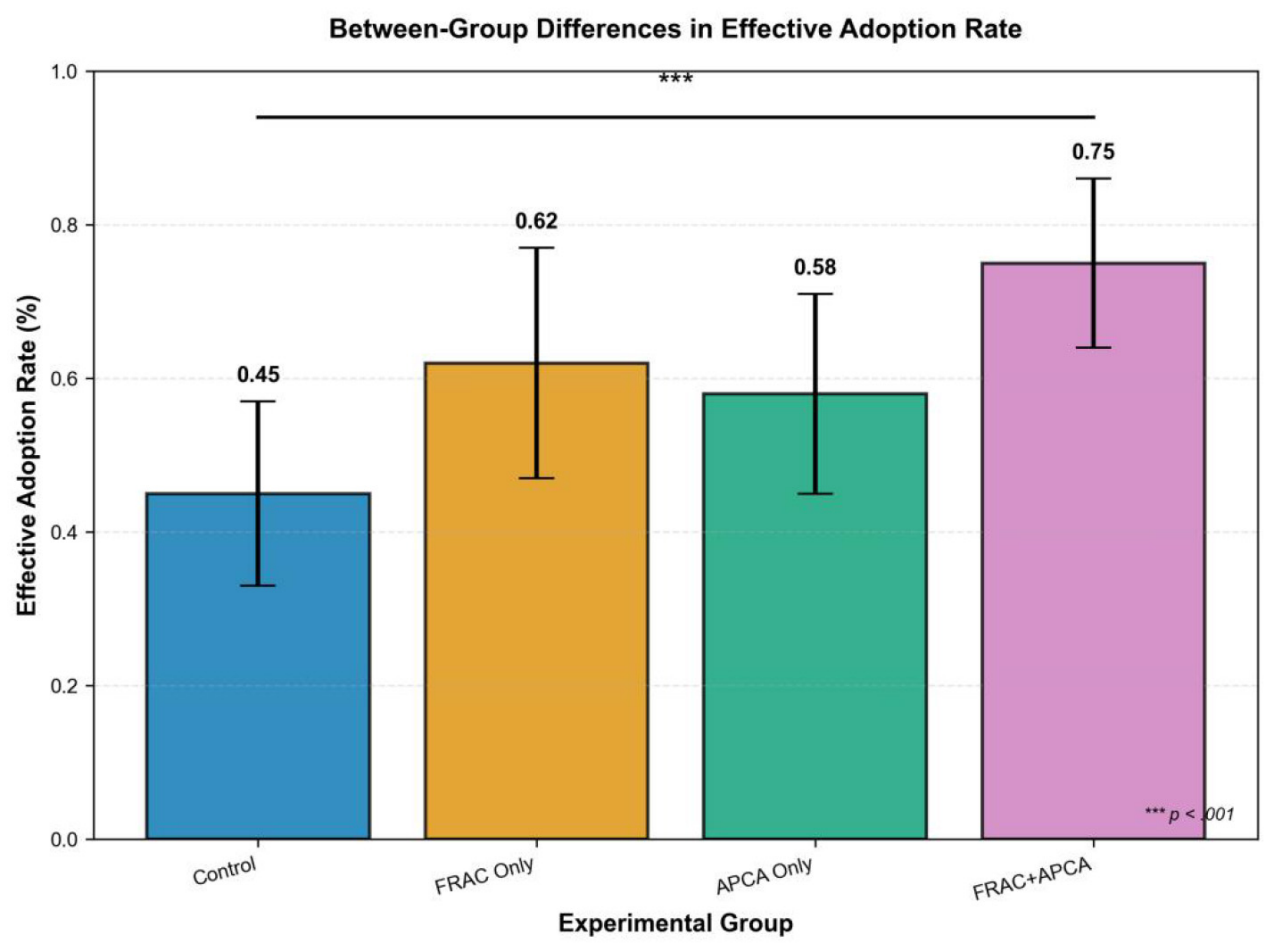
Visual comparison of effective adoption rates. ^***^p < 0.001.

As shown in Figure 6, the four groups differed substantially in effective adoption rate. The combined group obtained the highest mean score (M = 0.75), followed by the FRAC group (M = 0.62), the APCA group (M = 0.58), and the regular AI group (M = 0.45), and the overall group difference was significant (*p* < 0.001). Compared with the regular AI group, all three intervention groups converted AI feedback into effective revisions more successfully, with the advantage being most pronounced in the combined group. This result indicates that support for feedback processing and self-judgment, especially when provided together, was associated with more effective use of AI feedback, while the higher score of the FRAC group further suggests that feedback literacy support helped students assess and enact AI feedback more productively.

### 4.5 Distribution of revision depth

**Figure 7:**
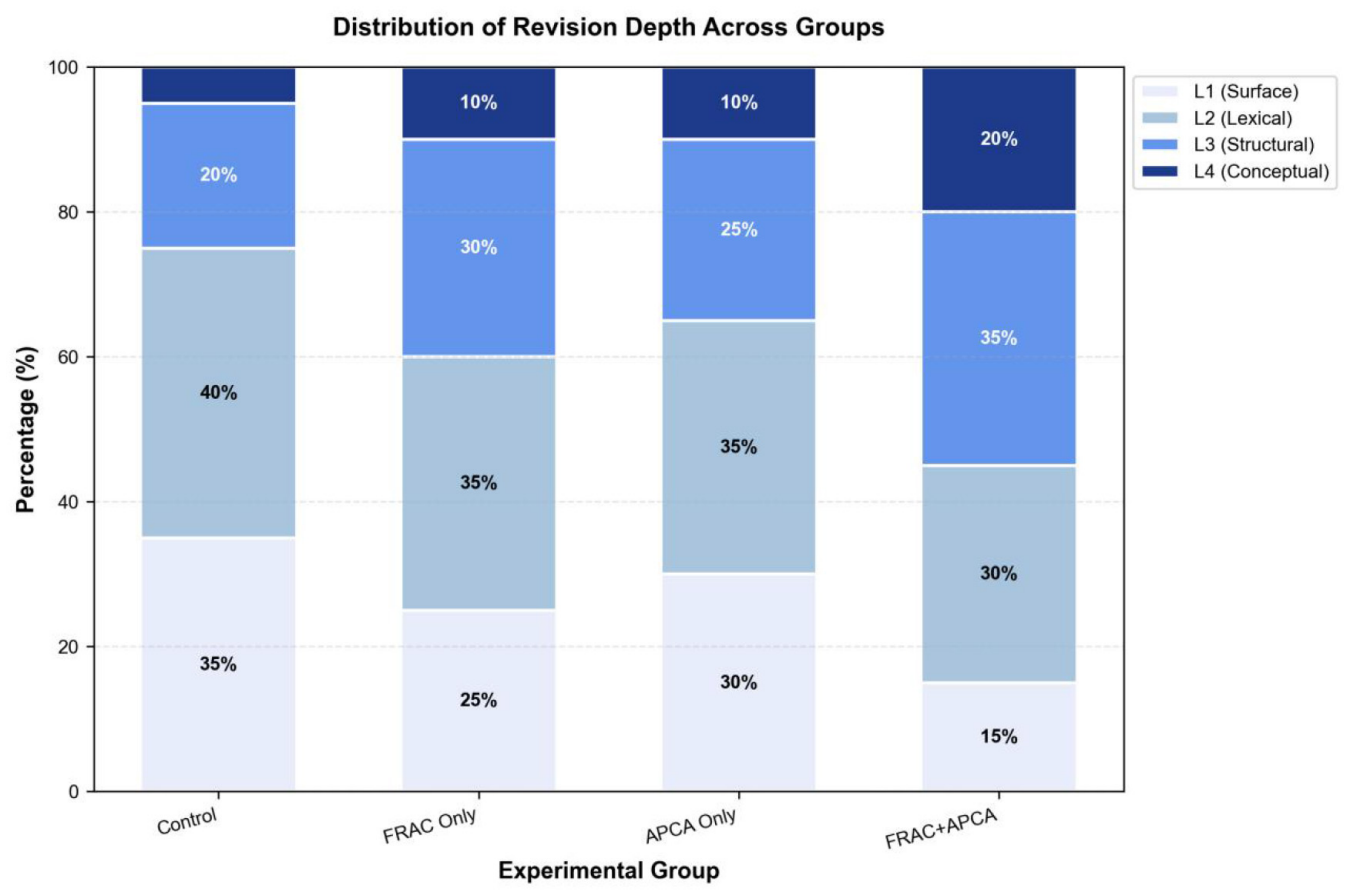
Distribution of revision depth across the four groups.

Figure 7 shows marked differences in revision depth across the four groups. Revision in the regular AI group was concentrated at the shallow levels, with 35% at L1 and 40% at L2, whereas only 20% and 5% reached L3 and L4, indicating that most changes were limited to surface correction and local language adjustment. The APCA group followed a broadly similar pattern (30%, 35%, 25%, and 10% from L1 to L4), although it showed a modest shift toward deeper revision than the regular AI group. A different pattern appeared in the FRAC and combined groups. In the FRAC group, L3 and L4 accounted for 30% and 10% of revisions, and in the combined group these proportions increased further to 35% and 20%. When L3 and L4 were considered together, the share of deep revision rose from 25% in the regular AI group to 35% in the APCA group, 40% in the FRAC group, and 55% in the combined group. Taken together, these results indicate that FRAC was more closely associated with structural and conceptual revision, and that the combined condition was linked to the greatest shift toward deeper revision.

### 4.6 Transfer and retention effects

**Figure 8:**
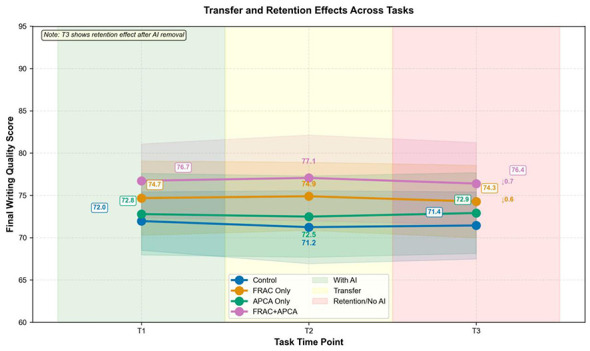
Performance of the four groups in the transfer task and retention task.

Figure 8 shows that final draft scores changed little from T1 to T3, and the ordering of the four groups was broadly stable across the three tasks. The combined group remained highest throughout, followed by the FRAC group, the APCA group, and the regular AI group. At T2, when the writing topic shifted, the FRAC and combined groups maintained their performance without an evident drop, indicating that the advantage associated with these two conditions was not confined to the original task. A similar pattern appeared at T3, which was completed without AI support: although scores fluctuated slightly across groups, the combined and FRAC groups still outperformed the APCA and regular AI groups. Taken together, these patterns point to some degree of transfer across tasks and retention after the removal of AI support, particularly in the FRAC and combined conditions.

## 5 Discussion

This study examined the effects of two forms of metacognitive support, FRAC and APCA, on students' writing performance in an AI-assisted writing context. The findings suggest that AI feedback does not automatically lead to writing improvement; rather, its value depends on whether students can evaluate, select, and transform feedback into meaningful revision decisions. This interpretation is consistent with feedback literacy research, which emphasizes that feedback becomes useful only when learners understand, judge, and act on feedback information (Molloy et al., 2020). In AI-assisted writing, this issue is particularly important because AI feedback is often immediate and abundant, but students still need to decide which suggestions are relevant, accurate, and worth adopting. The stronger performance of the FRAC group therefore suggests that feedback literacy support helped students move from feedback reception to feedback enactment. This finding extends previous work on automated and generative AI feedback by showing that students' engagement with feedback, rather than feedback availability alone, is central to learning from AI-supported writing tools (Ranalli, 2021; Zhan and Yan, 2025).

The results for self-assessment accuracy showed a different pattern. After baseline differences were controlled, APCA had the clearest positive effect, whereas FRAC did not significantly improve this outcome. This finding can be explained through research on self-assessment and metacognitive monitoring. Self-assessment is a core component of self-regulated learning because it helps learners judge the quality of their own work, identify performance gaps, and adjust subsequent learning actions (Panadero and Alonso-Tapia, 2013; Andrade, 2019; Yan, 2020). APCA was designed to make students compare perceived performance with actual performance, and this comparison may have directly supported calibration by reducing judgment error and overconfidence. The finding also aligns with studies showing that accurate self-assessment requires explicit training and does not necessarily develop from feedback exposure alone (Brown et al., 2015; Kostons et al., 2012; Panadero et al., 2017). In this sense, APCA mainly strengthened judgment calibration, whereas FRAC mainly supported feedback use and textual revision.

The different effects of FRAC and APCA indicate that writing improvement and judgment accuracy, although related, are not identical developmental outcomes. This distinction is consistent with theories of metacognition and self-regulated learning, which differentiate among monitoring, control, and strategy-use processes (Dinsmore, 2017; Panadero, 2017; Winne and Hadwin, 1998). APCA appears to have supported monitoring and calibration, while FRAC appears to have supported control and enactment during revision. This distinction helps explain why the combined group achieved the strongest writing performance but did not outperform the APCA-only group in self-assessment accuracy. The process indicators further support this interpretation: students receiving FRAC, especially in the combined group, showed higher effective adoption rates and a larger proportion of deep revisions. Because revision involves not only surface editing but also planning, evaluation, restructuring, and reformulation of meaning (Flower and Hayes, 1981), the increase in deep revision suggests that FRAC helped students use AI feedback for more substantive textual change.

The transfer and retention findings further suggest that the benefits of FRAC were not limited to immediate AI-supported revision. The FRAC and combined groups maintained their advantage in the transfer task and continued to perform better in the retention task after AI support had been withdrawn. This pattern suggests that structured feedback-processing support may help students internalize revision strategies and apply them beyond the original task context. This interpretation is consistent with self-regulated learning research, which argues that strategy training can support transfer when learners are guided to monitor task demands and regulate their learning actions across contexts (Winne and Hadwin, 1998; Wirth et al., 2025). At the same time, the smaller advantage at T3 indicates that AI feedback still contributed to immediate revision quality. Thus, the study supports a balanced view of AI-assisted writing: AI tools can provide useful feedback, but their learning value is more likely to be realized when students are supported in making independent judgments about that feedback (Kasneci et al., 2023; Henderson et al., 2025).

From a pedagogical perspective, these findings suggest that AI-assisted writing instruction should not remain at the level of merely providing feedback. Without guidance, students may prioritize surface-level suggestions that are easier to implement and may pay less attention to higher-order issues such as argument structure, coherence, and idea development. Feedback literacy support and calibration training can provide complementary forms of metacognitive support: the former helps students process external feedback more critically and productively, while the latter helps students judge their own performance more accurately. Together, these forms of support may reduce passive dependence on AI and encourage students to remain active decision-makers in the revision process. Nevertheless, this study has several limitations. The sample was limited to English majors from one university, the APCA group showed lower raw self-assessment error at baseline, and effective adoption rate was measured directly only in the FRAC and combined groups. Future research should test the intervention in more diverse contexts, collect comparable process data across all groups, and incorporate variables such as trust in AI feedback, perceived effort, learner agency, and critical AI literacy to clarify the mechanism linking AI feedback use and writing improvement (Chan and Hu, 2023).

## 6 Conclusion

This study discussed the role of structured metacognitive support in students' use of AI feedback for writing revision. By comparing the FRAC, the APCA, and their combined form, the study found that whether AI feedback can be translated into writing improvement depends not only on the generation of feedback itself, but also on whether students receive support in understanding, judging, and acting on that feedback. In terms of the different interventions, FRAC was more clearly associated with improved writing quality and deeper revision, whereas APCA was more closely reflected in improved self-assessment accuracy and reduced overconfidence. When the two were combined, students showed stronger overall writing gains and better retention, suggesting that feedback-processing support and calibration support do not replace each other but can complement one another in the revision process.

The main significance of this study lies in shifting the focus from “whether AI can provide feedback” to “under what conditions AI feedback can genuinely support learning.” The results indicate that changes in students' writing performance are not determined primarily by the amount of feedback or the efficiency of feedback generation, but are more closely related to whether students can judge, select, and form independent revision decisions based on that feedback. This also suggests that, when AI writing tools are used in teaching, they should not be treated merely as sources of instant feedback. Instead, they should be embedded within specific instructional support structures, so that students can develop independent judgment and self-regulated learning abilities while using feedback. Future research may further examine the relationships among feedback literacy, calibration ability, learner agency, and trust in AI in order to explain the internal mechanisms of writing development under AI-supported conditions in greater detail.

## Data Availability

The raw data supporting the conclusions of this article will be made available by the authors, without undue reservation.
